# Genetic determinants of serum vitamin B12 and their relation to body mass index

**DOI:** 10.1007/s10654-016-0215-x

**Published:** 2016-12-19

**Authors:** Kristine H. Allin, Nele Friedrich, Maik Pietzner, Niels Grarup, Betina H. Thuesen, Allan Linneberg, Charlotta Pisinger, Torben Hansen, Oluf Pedersen, Camilla H. Sandholt

**Affiliations:** 1 The Novo Nordisk Foundation Center for Basic Metabolic Research, Section of Metabolic Genetics, Faculty of Health and Medical Sciences, University of Copenhagen, Universitetsparken 1, 2100 Copenhagen, Denmark; 2 Institute of Clinical Chemistry and Laboratory Medicine, University Medicine Greifswald, Greifswald, Germany; 3 Research Centre for Prevention and Health, The Capital Region of Denmark, Copenhagen, Denmark; 4 Department of Clinical Experimental Research, Rigshospitalet, Glostrup, Denmark; 5 Faculty of Health Sciences, University of Southern Denmark, Odense, Denmark; 6 Faculty of Health Sciences, University of Aarhus, Aarhus, Denmark

**Keywords:** BMI, Cobalamin, *FUT2*, Mendelian randomization, Secretor/nonsecretor, Vitamin B12

## Abstract

Lower serum vitamin B12 levels have been related to adverse metabolic health profiles, including adiposity. We used a Mendelian randomization design to test whether this relation might be causal. We included two Danish population-based studies (n_total_ = 9311). Linear regression was used to test for associations between (1) serum vitamin B12 levels and body mass index (BMI), (2) genetic variants and serum vitamin B12 levels, and (3) genetic variants and BMI. The effect of a genetically determined decrease in serum vitamin B12 on BMI was estimated by instrumental variable regression. Decreased serum vitamin B12 associated with increased BMI (*P* < 1 × 10^−4^). A genetic risk score based on eight vitamin B12 associated variants associated strongly with serum vitamin B12 (*P* < 2 × 10^−43^), but not with BMI (*P* = 0.91). Instrumental variable regression showed that a 20% decrease in serum vitamin B12 was associated with a 0.09 kg/m^2^ (95% CI 0.05; 0.13) increase in BMI (*P* = 3 × 10^−5^), whereas a genetically induced 20% decrease in serum vitamin B12 had no effect on BMI [−0.03 (95% CI −0.22; 0.16) kg/m^2^] (P = 0.74). Nevertheless, the strongest serum vitamin B12 variant, *FUT2* rs602662, which was excluded from the B12 genetic risk score due to potential pleiotropic effects, showed a per allele effect of 0.15 kg/m^2^ (95% CI 0.01; 0.32) on BMI (*P* = 0.03). This association was accentuated including two German cohorts (n_total_ = 5050), with a combined effect of 0.19 kg/m^2^ (95% CI 0.08; 0.30) (*P* = 4 × 10^−4^). We found no support for a causal role of decreased serum vitamin B12 levels in obesity. However, our study suggests that FUT2, through its regulation of the cross-talk between gut microbes and the human host, might explain a part of the observational association between serum vitamin B12 and BMI.

**Electronic supplementary material:**

The online version of this article (doi:10.1007/s10654-016-0215-x) contains supplementary material, which is available to authorized users.

## Introduction

Human obesity is a highly heterogeneous disorder where multiple factors, especially related to lifestyle, contribute to the pathogenic development. A high degree of heritability has also been established [1], and a substantial number of genetic variants associated with obesity-related phenotypes have been identified [2, 3]. The pathways and mechanisms underlying these genetic associations have not been fully elucidated, but central nervous system and adipose tissue biology have been highlighted. Nevertheless, the genetic variants only explain a small fraction of the phenotypic variance [2, 3], indicating that yet undiscovered mechanisms could be essential in the obesity pathogenesis.

Vitamin B12 (cobalamin) is solely produced by Bacteria and Archaea [4] and the only natural source of this vitamin for humans are foods of animal origin such as meat, fish, eggs, and dairy products. In humans, vitamin B12 functions as an essential coenzyme in various cellular functions such as cellular energy metabolism, DNA synthesis and methylation, as well as protein, lipid, and carbohydrate synthesis [5]. Several studies have observed associations between lower circulating vitamin B12 levels and adverse metabolic health profiles, with insulin resistance, cardiovascular disorders, and adiposity as important features [6–10].

The heritability of serum vitamin B12 levels has in a study of mono- and dizygotic twins been estimated to be 59% [11]. Several studies have, in genome-wide association studies (GWAS) and using sequencing efforts, identified at least 12 genetic variants robustly associated with circulating vitamin B12 levels [12–16]. Interestingly, despite using hypothesis-free genome-wide approaches, the vast majority of the identified single nucleotide polymorphisms (SNP) are situated in or near genes encoding proteins responsible for the absorption, processing, and coenzymatic activity of vitamin B12 [16].

These genetic variants allow the construction of a genetic risk score (GRS) that can serve as a relatively unbiased instrument for serum vitamin B12 levels in a Mendelian randomization study [17]. Here, we used this instrument in two Danish population-based cohorts conducted at a time point where legislation did not allow fortification with vitamin B12 (or folate) of food items [18], to test the hypothesis that lower serum vitamin B12 levels are causally related to obesity.

## Materials and methods

### Study populations

The Danish population-based Inter99 study (ClinicalTrials.gov ID-no: NCT00289237) is a non-pharmacological intervention study for ischemic heart disease, initiated in 1999 at the Research Centre for Prevention and Health, Glostrup, Denmark. A random sample of 13,016 individuals living in Copenhagen County from seven different age groups (30–60 years, grouped with 5 year intervals) was drawn from the Civil Registration System and 6784 of these attended the health examination [19].

The Danish Health2006 cohort is a population-based study of general health and chronic diseases such as type 2 diabetes and cardiovascular disease in individuals aged 18–69 years. The study was initiated in 2006 at Research Centre for Prevention and Health, Glostrup, Denmark, and participants were drawn as a random sample of the background population living in the south-western part of the greater Copenhagen area. A total of 7770 were invited to participate and 3471 entered and participated in the health examination [20].

The Study of Health in Pomerania (SHIP-0) is a population-based study in West Pomerania, a rural region in north-east Germany [21], where 6265 individuals, aged 20–79 years, were invited, of which 4308 participated. Data collection was performed between 1997 and 2001. For SHIP-TREND, an independent random sample of 8826 individuals aged 20–79 years was drawn from the same region between 2008 and 2012, and 4420 participated.

All participants gave written informed consent. The studies were approved by local ethics committees and conformed to the principles of the declaration of Helsinki.

### Anthropometric, biochemical, and lifestyle measures

In all cohorts, body weight (kg), waist circumference (cm), hip circumference (cm) and height (cm) were measured in light indoor clothes and without shoes. Body mass index (BMI) was calculated as body weight divided by the squared height in meters (kg/m^2^). In the Health2006 cohort body fat percentage was measured using bioimpedance.

For the Danish cohorts, vitamin B12 was measured in serum obtained from fasting blood samples, stored at −20 °C until the analyses were performed in 2008. For the German cohorts, blood samples were analyzed immediately or stored at −80° until analysis. In all cohorts, serum vitamin B12 was measured using a competitive chemiluminescent enzyme immunoassay (CENTAUR or Dimension Vista platform, Siemens Healthcare Diagnostics), performed by Institute of Clinical Chemistry and Laboratory Medicine, University Medicine Greifswald, Germany.

In the Danish cohorts, all lifestyle factors were estimated from self-reported questionnaire data. A three-point dietary score was developed based on a 48-item food frequency questionnaire and categorized as; (1) unhealthy, (2) moderately healthy, and (3) healthy [22]. Alcohol intake was estimated as the total units of alcohol (1 unit equals 12 grams of alcohol) per week. The level of physical activity was estimated summing the time spent actively commuting (min/week) and time spent on leisure time physical activity (min/week). Subsequently, four categories were created; (1) 0–2 h/week, (2) 2–4 h/week, (3) 4–7 h/week, and (4) 7–12 h/week [23]. Smoking habits were divided into three classes: (1) never smoked, (2) former smoker, (3) daily and occasional smoker [24].

### Genotyping, SNP selection, and genetic risk score

Genotyping is described in detail in Supplemental Materials and Methods. Inter99 and Health2006 were genotyped using the Human Exome BeadChip on an Illumina HiScan system (Illumina). In Inter99 a total of 6161 individuals were genotyped and passed all quality control criteria and the corresponding number was 2914 for Health2006. The average call rate for all SNPs on the Human Exome BeadChip was 99.0%.

The SNPs for the GRS were selected based on a joint Icelandic and Danish sequencing initiative reporting 11 loci associated genome-wide significantly (*P* < 5 × 10^−8^) with serum vitamin B12 levels [16], of which six were validation of previous GWAS in European and Chinese populations [12–15]. Of the 11 serum vitamin B12 associated SNPs [16], ten (*MMAA* rs2270655, *MUT* rs1141321, *CUBN* rs1801222, *TCN1* rs34324219, *CLYBL* rs41281112, *ABCD4* rs3742801, *CD320* rs2336573, *TCN2* rs1131603, *FUT6* rs778805 and *FUT2* rs602662) could be retrieved from the Human Exome BeadChip, whereas one (*MMACHC* rs12272669) was not represented. The SNP quality of the ten SNPs retrieved from the Human Exome BeadChip was estimated based on genotype call rate (>95%), Hardy–Weinberg equilibrium (HWE) (*P* > 0.005), or cross-hybridization with the X-chromosome, and all ten SNPs passed these filters (HWE; *P* > 0.04, call rate >98.1%).

The genotypes were coded according to number of serum vitamin B12 decreasing alleles. For four SNPs the minor allele, and for six SNPs the major allele, were considered the effect allele which had frequencies ranging from 3.0 to 96.9% in Inter99 and from 3.0 to 97.2% in Health2006. A full vitamin B12 GRS (full-B12 GRS) was calculated by summarizing the number of serum vitamin B12 decreasing alleles for the ten SNPs for each individual. Additionally, a B12 GRS without *FUT2* rs602662 and *FUT6* rs778805 (B12 GRS) was constructed to circumvent the pleiotropic effects of *FUT2* and *FUT6*.

SHIP-0 was genotyped using the Affymetrix Genome-Wide Human SNP Array 6.0. *FUT2* rs602662 was imputed based on the 1000G project (phase 1, European super-population, March 2012 release [25]) with an imputation quality score of 0.99. Genotype data were available for 4064 subjects. Only a subset of SHIP-TREND (n = 986) was genotyped. This was done using the Illumina Human Omni 2.5 array, and rs602662 was directly genotyped with a callrate of 99.8% obeying HWE (P = 0.02).

### Statistical analyses

The statistical analyses were performed using the statistical software R version 3.1.1 (http://www.r-project.org/) and Stata (version 13.1; StataCorp). *P* values below 0.05 were considered statistically significant. Linear regression models (adjusted for age and sex) were used to test for association between (1) serum vitamin B12 levels and BMI, (2) SNPs or B12 GRS and serum vitamin B12 levels, and (3) SNPs or B12 GRS and BMI. The serum vitamin B12 and BMI association was additionally tested using multifactor adjustment (diet, alcohol consumption, physical activity, and smoking). Association between the B12 GRS and potential confounders were tested using linear regression models for continuous outcomes (age and alcohol consumption) and logistic regression for categorical outcomes (diet, physical activity, and smoking). Associations between individual SNPs or the B12 GRS and serum vitamin B12 levels/BMI were analyzed using an additive model. The *FUT2* rs602662 genotype was additionally analyzed using a dominant model. Explained phenotypic variance (R^2^) was estimated using unadjusted linear models. The effect of a genetically determined decrease in serum vitamin B12 on BMI was estimated using unadjusted instrumental variable regressions. These were performed using the ivreg function of the ‘AER’ package in R and the ivregress command in Stata, both using two-stage least-squares regression. The strengths of the instruments were evaluated by F-statistics from first stage regressions. In addition to using the B12 GRS as an instrument, we also used each of the variants as separate instruments, which is equivalent to using a GRS where each variant is weighted based on its association with serum vitamin B12 [26]. To test for directional pleiotropy and to evaluate the robustness of our findings, we also applied MR-Egger regression. Pleiotropy was evaluated by the MR-Egger test which tests whether the intercept differs from zero and by visually inspecting Funnel plots for asymmetry [26]. The combined effect across study populations was estimated using fixed-effect model meta-analyses performed by the rma function of the ‘metafor’ package in R. Linkage disequilibrium was estimated using SNAP (http://www.broadinstitute.org/mpg/snap/) in data from the European panel of HapMap phase 3 data.

Data on BMI from the Genetic Investigation of Anthropometric Traits (GIANT) consortium was obtained from the European ancestry dataset (http://www.broadinstitute.org/collaboration/giant/index.php/GIANT_consortium_data_files).

## Results

The phenotypic characteristics of the two Danish cohorts, Inter99 and Health2006, are shown in Supplementary Table 1. Individuals were middle-aged, with a median BMI of 25.6 and 25.2 kg/m^2^, respectively. Serum vitamin B12 levels were higher in Health2006 compared to Inter99, but similar among women and men within cohorts (Supplemental Table 1).

### Observational association between serum vitamin B12 levels and BMI

**Fig. 1:**
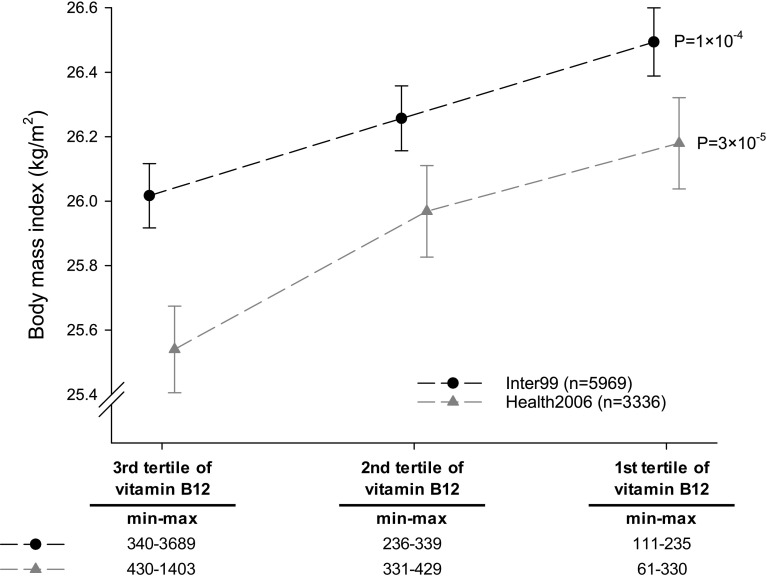
Observational association between serum vitamin B12 levels divided in tertiles and BMI. *Dots* and *triangles* indicate mean BMI and error bars represent standard errors. *Black dots* represent Inter99 and *grey triangles* represent Health2006. *P* values are from age and sex adjusted linear regression of BMI on serum vitamin B12 levels

Decreasing levels of serum vitamin B12 were associated with increasing BMI in the two Danish cohorts (Fig. 1). A BMI difference of 0.48 (95% CI 0.19; 0.76) kg/m^2^ and 0.64 (95% CI 0.26; 1.02) kg/m^2^ was observed when comparing the first and third tertile of serum vitamin B12 levels in Inter99 and Health2006, respectively. Accordingly, a 20% decrease in serum vitamin B12 levels was associated with an increase in BMI of 0.10 (95% CI 0.05; 0.14) kg/m^2^ (*P* = 1 × 10^−4^) in Inter99 and 0.19 (95% CI 0.10; 0.27) kg/m^2^ (*P* = 3 × 10^−5^) in Health2006. Linear regression model additionally adjusted for lifestyle factors (diet, alcohol consumption, physical activity, and smoking), as well as exclusion of type 2 diabetes patients receiving anti-diabetic treatment (n_Inter99_ = 71, n_Health2006_ = 66) showed similar results (Supplemental Table 2).

In addition to BMI, decreasing levels of serum vitamin B12 were associated with increasing waist circumference and body fat percentage. A 20% decrease in serum vitamin B12 levels was associated with an increase in waist circumference of 0.23 (95% CI 0.11; 0.35) cm (*P* = 3 × 10^−4^) in Inter99 and 0.46 (95% CI 0.24; 0.69) cm (*P* = 6 × 10^−5^) in Health2006. Body fat percentage was only measured in Health2006, where a 20% decrease in serum vitamin B12 levels was associated with an increase in body fat percentage of 0.29 (95% CI 0.16; 0.42)% (*P* = 1 × 10^−5^). Decreasing levels of serum vitamin B12 were also associated with increasing waist/hip ratio in Health2006 (*P* = 0.03) but not in inter99 (*P* = 0.14).

### Genotypes and serum vitamin B12 levels

The ten SNPs included in the full-B12 GRS each associated with decreased serum vitamin B12 levels in both Inter99 and Health2006 (Supplemental Table 3). The SNP showing the strongest association was *FUT2* rs602662 with a per G-allele decrease in serum vitamin B12 of −10 (95% CI −11; −8)% (*P* = 3 × 10^−37^) according to an additive model in Inter99. However, the median serum vitamin B12 levels suggested that a dominant model would be more appropriate. Comparing homozygous/heterozygous G-allele carriers with homozygous A-allele carriers showed a serum vitamin B12 level decrease of −20 (95% CI −22; −17)% (*P* = 1 × 10^−51^). A similar association pattern was observed in Health2006 (Supplemental Table 3).

**Fig. 2:**
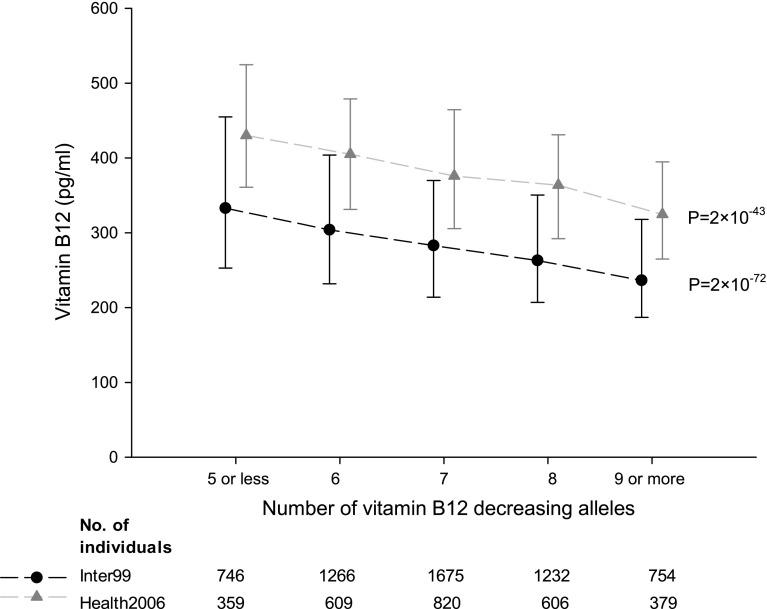
Association between the B12 GRS and serum vitamin B12 levels. Vitamin B12 decreasing alleles ranged from 2 to 13. Individuals with ≤5 or ≥9 alleles were grouped to obtain a reasonable number of individuals in each group. *Dots* and *triangles* indicate median serum vitamin B12 and *error bars* indicate interquartile range. *P* values are from age and sex adjusted linear regression

The full-B12 GRS associated strongly with serum vitamin B12 levels, with a per effect allele decrease of −7 (95% CI −8; −7)% (*P* = 5 × 10^−104^) in Inter99, and a similar association was observed in Health2006 (Supplemental Table 4). However, the overall aim of constructing a GRS was to obtain an instrument for serum vitamin B12 levels not influenced by potential confounders. FUT2 and FUT6 are known to determine ABH antigen secretor/nonsecretor status and be involved in the creation of Lewis antigens, respectively. Hence, both could have pleiotropic effects, and therefore, we also analyzed a GRS excluding *FUT2* rs602662 and *FUT6* 778805 (B12 GRS). The B12 GRS was not associated with potential confounders including age, sex, diet, alcohol consumption, physical activity, and smoking (Table 1). The proportion of physically inactive individuals was seemingly different among the B12 GRS allele groups, however, the direction of effect was opposite in the two cohorts, which may suggest that this is a chance finding (type 1 error). The B12 GRS showed a per allele decrease in serum vitamin B12 of −7 (95% CI −8; −7)% (*P* = 2 × 10^−72^) in Inter99, and −6 (95% CI −7; −5)% (*P* = 2 × 10^−43^) in Health2006 (Fig. 2; Supplemental Table 4). The F-statistics for the models were 331 in Inter99 and 197 in Health2006. The B12 GRS explained 5.5 and 6.7% of the phenotypic variance in serum vitamin B12 in Inter99 and Health2006, respectively (Supplemental Table 4).

**Table 1:** Association between the B12 GRS and potential confounders

	Number of vitamin B12 decreasing alleles					
Inter99	5 or less (n = 785)	6 (n = 1377)	7 (n = 1797)	8 (n = 1313)	9 or more (n = 809)	*P*_trend_
Women, n (%)	420 (54)	678 (50)	928 (52)	647 (49)	431 (53)	0.39
Age, years	45 (40–50)	45 (40–50)	45 (40–50)	45 (40–55)	45 (40–50)	0.75
Unhealthy diet, n (%)^a^	117 (16)	195 (15)	278 (16)	210 (17)	126 (16)	0.52
Alcohol consumption, units per week^b^	7 (3–14)	7 (2–14)	7 (2–14)	6 (2–14)	6 (3–14)	0.93
Physical inactivity, n (%)^c^	269 (36)	452 (35)	598 (36)	408 (33)	239 (32)	0.07
Current smokers, n (%)^d^	330 (43)	507 (37)	706 (40)	517 (40)	319 (40)	0.23

Health2006	5 or less (n = 370)	6 (n = 630)	7 (n = 842)	8 (n = 624)	9 or more (n = 391)	*P*_trend_
Women, n (%)	200 (54)	342 (54)	446 (53)	348 (56)	224 (57)	0.49
Age, years	49 (41–60)	51 (40–61)	51 (41–60)	50 (41–60)	50 (40–60)	0.52
Unhealthy diet, n (%)^a^	24 (7)	44 (7)	53 (6)	43 (7)	23 (6)	0.89
Alcohol consumption, units per week^b^	6 (2–13)	7 (2–14)	6 (2–13)	6 (2–12)	6 (2–14)	0.25
Physical inactivity, n (%)^c^	146 (40)	275 (45)	388 (46)	304 (49)	189 (49)	0.003
Current smokers, n (%)^d^	95 (26)	167 (27)	202 (24)	173 (28)	98 (25)	0.87

Values are median (IQR) unless otherwise specified. *P*_trend_ are from linear regressions for continuous potential confounders (age and alcohol) and from logistic regressions for categorical potential confounders: ^a^ Unhealthy diet vs. moderately healthy and healthy diet; ^b^ 1 unit = 12 g of alcohol; ^c^ Physical activity <4 h per week versus ≥4 h per week; ^d^ Current smokers versus former and never smokers

### B12 GRS and BMI

If decreased levels of serum vitamin B12 *cause* increased BMI, the B12 GRS would, given its strong association with serum vitamin B12 levels, expectedly also associate with increased BMI. However, we observed no association between the B12 GRS and BMI in either Inter99 or Health2006. A fixed-effect model was used to combine the effect sizes from the two cohorts and resulted in a per allele effect of 0.00 (95% CI −0.07; 0.06) kg/m^2^ (*P* = 0.91) (Table 2). Accordingly, when analyzed separately, none of the eight SNPs included in the B12 GRS associated with BMI in the two cohorts (data not shown).

**Table 2:** Association between the B12 GRS and BMI

	n	Weight (%)	Per allele effect, kg/m^2^ (95% CI)	*P* value
B12 GRS				
Inter99	6076	68.5	−0.03 (−0.11;0.06)	
Health2006	2854	31.5	0.04 (−0.08;0.17)	
Fixed-effect model			0.00 (−0.07;0.06)	0.91
Heterogeneity: I^2^ = 0.0%, *P* = 0.35				

Per allele effects are from linear regression analyses of the B12 GRS on BMI adjusted for age and sex

### Observational versus genetic associations

**Fig. 3:**
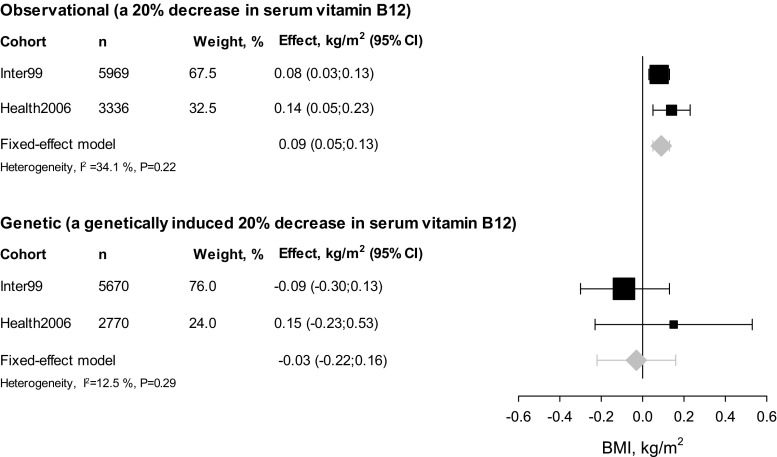
Association between serum vitamin B12 levels and BMI. *Squares* indicate effect estimates from Inter99 and Health2006, sized according to the weight of each study in the meta-analyses. Diamonds indicate effect estimates from fixed-effects meta-analysis and *error bars* indicate 95% CI

To directly compare the observational effect of decreased serum vitamin B12 levels on BMI with the effect of genetically induced low serum vitamin B12 levels on BMI, we performed an instrumental variable analysis. The combined observational effect of a 20% decrease in serum vitamin B12 on BMI in the two cohorts was an increase of 0.09 (95% CI 0.05; 0.13) kg/m^2^, (*P* = 3 × 10^−5^), whereas a genetically induced 20% decrease in serum vitamin B12 had no effect on BMI (−0.03 (95% CI −0.22; 0.16) kg/m^2^, *P* = 0.74). Since we observed an association between decreased serum vitamin B12 levels and BMI but no association between genetically induced decreased serum vitamin B12 levels and BMI, the present results do not support a causal role of serum vitamin B12 levels on BMI. However, as the observational estimate (0.09) is included in the 95% CI of the genetic estimate (−0.22; 0.16), we cannot completely rule out causality (Fig. 3).

### FUT2 genotype and BMI

We hypothesized that *FUT2* rs602662, through yet undescribed pleiotropic effects, could explain a portion of the observational effect between serum vitamin B12 and BMI given the strong association with serum vitamin B12 (Supplemental Table 3). The serum vitamin B12 decreasing *FUT2* rs602662 G-allele associated with increased BMI when combining Inter99 and Health2006 in a fixed-effects meta-analysis (0.15 (95% CI 0.01; 0.32) kg/m^2^, *P* = 0.03), and the *FUT2* genotype explained 0.05 and 0.02% of the phenotypic variance in BMI in Inter99 and Health2006, respectively.

### Validation analyses in two German cohorts

**Fig. 4:**
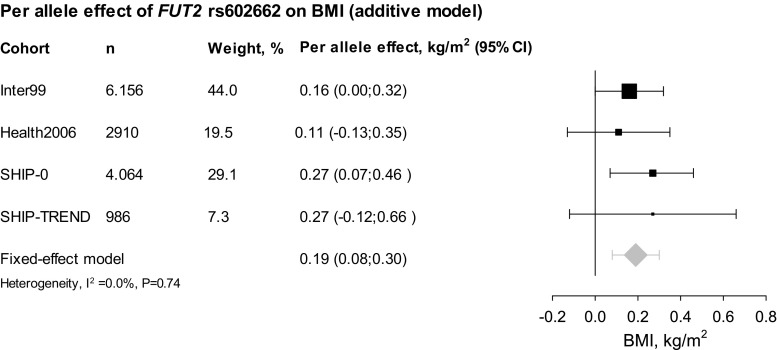
Association between *FUT2* rs602662 and BMI. *Squares* indicate effect estimates from Inter99 and Health2006, sized according to the weight of each study in the meta-analyses. The *diamonds* indicates the effect estimate from fixed-effects meta-analysis and *error bars* indicate 95% CI

Individuals in the German cohorts were middle-aged with slightly higher BMI than in Inter99 and Health2006 with a median BMI of 26.8 and 27.5 kg/m^2^ for SHIP-0 and SHIP-TREND, respectively (Supplemental Table 5). Serum vitamin B12 levels were not significantly associated with BMI (data not shown), but the serum vitamin B12 decreasing *FUT2* rs602662 G-allele associated with increased BMI when combining SHIP-0 and SHIP-TREND in a fixed-effects meta-analysis [0.27 (95% CI 0.09; 0.44) kg/m^2^, *P* = 0.003]. The *FUT2* rs602662 genotype explained 0.12% of the phenotypic variance of BMI in both SHIP-0 and SHIP-TREND. When combining the Danish and the German cohorts, the *FUT2* rs602662 showed a combined per G-allele effect of 0.19 (95% CI 0.08; 0.30) kg/m^2^, *P* = 4 × 10^−4^ on BMI (Fig. 4). This association was supported by the European BMI data from the GIANT consortium where the *FUT2* rs602662 G-allele (n = 233,871) associated nominally with increased BMI (P = 0.01).

### Sensitivity analyses

Results from instrumental variable analysis where each of the eight SNPs were used as separate instruments (equivalent to a GRS weighted based on the SNP-serum vitamin B12 association) were similar to results from instrumental variable analysis based on the simple allele-counting B12 GRS (Supplemental Table 6; Fig. 3). Causal estimates from MR-Egger regression were largely similar to two-stage least-squares regression estimates although the estimates from MR-Egger regression were underpowered (Supplemental Table 5; Supplemental Fig. 1). This was the case both when including and excluding *FUT2* rs602662 and *FUT6* rs778805. The intercept from MR-Egger regression did not differ from zero, neither when excluding *FUT2* rs602662 and *FUT6* rs778805, nor when including these two SNPs (Supplemental Table 6) suggesting that directional pleiotropy was not present. In support, Funnel plots were also largely symmetrical (Supplemental Fig. 1). No interaction was observed between the B12 GRS and BMI associated SNPs (Supplemental Table 7).

## Discussion

We found an observational association between decreased serum vitamin B12 levels and elevated BMI in two Danish cohorts. This led to the hypothesis that low serum vitamin B12 levels are causally involved in body mass regulation, and we investigated this by applying a Mendelian randomization design, including a formal instrumental variable analysis using a B12 GRS to model an unbiased genetically induced decrease of serum vitamin B12. However, our results provided no clear evidence for a causal role of low serum vitamin B12 levels in obesity.

The Mendelian randomization design applied in this study may be likened to a randomized clinical trial, where individuals are randomized by nature to carry genetic variants associating with low or high circulating levels of vitamin B12. The purpose of the randomization process in a clinical trial is to obtain an even distribution of potential confounders across the treatment arms, and likewise the Mendelian randomization design should result in an equal distribution of known and unknown confounders across genotypes. Additionally, reverse causality (here that higher BMI causes lower serum vitamin B12 levels) is circumvented since genotypes are non-modifiable. Hence, in the setting of a Mendelian randomization study, genotypes or a GRS are used as an unconfounded instrument for the risk factor under investigation and allows a largely unbiased evaluation of the causal role of the risk factor. To obtain a proper instrument for serum vitamin B12 levels, we constructed a GRS consisting of eight vitamin B12 decreasing SNPs in genes encoding proteins from the pathway responsible for the absorption, processing, and coenzymatic activity of vitamin B12 [16]. This B12 GRS associated strongly with serum vitamin B12 and accordingly explained ~6% of the phenotypic variance in serum vitamin B12 levels. For comparison, 97 BMI associated SNPs from the GIANT consortium cumulatively explain only 2.7% of the phenotypic variance in BMI [2], which among many reasons may be due to the unspecific representation of numerous different pathways where the variants are not directly linked functionally. The fact that the B12 GRS associated strongly and specifically with serum vitamin B12 levels and the fact that the eight SNPs included in the B12 GRS all have well-characterized roles in vitamin B12 functioning, are coding and thus potentially the true functional SNP, qualifies the B12 GRS as a useful instrument for evaluating causality. If serum vitamin B12 levels are causally linked to BMI, the B12 GRS should be related to BMI to the extent predicted by its influence on serum vitamin B12 levels. Although we did not have sufficient statistical power to completely rule out causality, our instrumental variable analyses provided no evidence that low serum vitamin B12 levels *cause* increased BMI.

A crucial assumption underlying the Mendelian randomization design is that the genotype is associated with the outcome only via the risk factor under investigation [17]. However, genetic variants may have pleiotropic effects and thus influence the outcome through other pathways than the one under investigation. To circumvent issues of pleiotropy, we therefore additionally applied the MR-Egger approach. Inclusion of *FUT2* rs602662 and *FUT6* rs778805 as instruments for genetically induced decreased serum vitamin B12 levels did not support introduction of directional pleiotropy, but the statistical power seemed to decrease markedly when applying the MR-Egger method. Nevetheless, *FUT2* rs602662 which showed the strongest individual SNP effect on serum vitamin B12, is *known* to have pleiotropic effects. Thus, our finding that the G-allele of *FUT2* rs602662 was associated with decreased levels of serum vitamin B12 as well as increased BMI, does not necessarily support a causal role of decreased vitamin B12 levels in obesity. This finding may be explained by the strong linkage disequilibrium (r^2^ = 0.81, D′ = 1.0) between *FUT2* rs602662 and *FUT2* rs601338 which encodes a nonsense mutation (W143X) causing a lack of presentation of blood group antigens A, B and H on mucosal surfaces and in secretions. This SNP could potentially modulate the composition and function of the gut microbiota, a hypothesis that is supported by various studies. Homozygous carriers of the stop codon (*FUT2* rs601338 AA carriers corresponding to *FUT2* rs602662 AA carriers) are nonsecretors and they have been shown to be resistant to infections with *Helicobacter pylori* [27, 28] and Norovirus, the major cause of viral acute gastroenteritis worldwide [29–32]. Infection with *H. pylori* may cause chronic gastritis [33] leading to decreased secretion of intrinsic factor and therefore impaired absorption of vitamin B12. Intriguingly, the resistance to gastrointestinal infections suggests that lack of blood group antigens on the intestinal mucosa not only inhibits the infectious potential of pathogenic gastrointestinal microbes, but also may inhibit the habitual interaction with commensal microbes in the gastrointestinal tract. This is supported by studies showing that secretor status influences the composition of the gut microbiota [34–36]. Also, several studies have reported a link between gut microbes and human obesity [37–39] and it has been demonstrated that germ-free mice are leaner than conventionally raised mice and that conventionalization of germ-free mice with microbiota from conventionally raised mice results in an increased body fat content [37]. Therefore, the slightly *lower* BMI observed among AA carriers of *FUT2* rs602662 (accompanied by *higher* serum vitamin B12 levels) in our study may be explained by the lack of expression of blood group antigens in the gastrointestinal tract and therefore impaired cross-talk with commensal microbes. In support of this interpretation, results from GIANT showed that the *FUT2* rs601338 A-allele (coding for the stop codon) associated nominally with decreased BMI (*P* = 0.007). However, mechanistic studies in rodents and statistically powered studies of humans involving characterization of host genotype, microbiota composition, and BMI, must be conducted to fully address this hypothesis.

In conclusion, we did not find support for a causal role of decreased serum vitamin B12 levels in obesity. However, our study gives rise to the hypothesis that the *FUT2* locus explains a part of the observational association between serum vitamin B12 and BMI through its regulation of the cross-talk between gut microbes and the human host. Furthermore, the B12 GRS constructed in this study may be used a valid instrument in future Mendelian randomization studies evaluating of the causal role of serum vitamin B12 levels in other disorders.

## Electronic supplementary material

Below is the link to the electronic supplementary material.

Supplementary material 1 (DOCX 97 kb)
