# Supplementary material for: Genetic determinants of serum vitamin B12 and their relation to body mass index
Source: Eur J Epidemiol. 2016 Dec 19;32(2):125–34. doi: 10.1007/s10654-016-0215-x (PMC5374184; doi:10.1007/s10654-016-0215-x)
Supplement: Supplementary file 1 — Supplementary material 1 (DOCX 97 kb) [file 10654_2016_215_MOESM1_ESM.docx]

**Genetic determinants of serum vitamin B12 and their relation to obesity**

Kristine H Allin*, Nele Friedrich, Maik Pietzner, Niels Grarup, Betina H Thuesen, Allan Linneberg, Charlotta Pisinger, Torben Hansen, Oluf Pedersen, and Camilla H Sandholt

*The NNF Center for Basic Metabolic Research

Faculty of Health and Medical Sciences, University of Copenhagen

Universitetsparken 1, DK-2100 Copenhagen Ø

Phone: +45 3533 7055

E-mail: kristine.allin@sund.ku.dk

**Supplemental materials and methods**

*Genotyping*

Inter99 and Health2006 were genotyped using the Human Exome BeadChip on an Illumina HiScan system (Illumina). Genotypes were called using the Genotyping module (version 1.9.4) of the GenomeStudio software (version 2011.1; Illumina) and using custom cluster data generated from 5,865 Danish DNA samples analyzed on the same Illumina HiScan. Individuals were excluded during quality control removing closely related individuals, individuals with an extreme inbreeding coefficient, individuals with a low callrate (<90%), individuals with a mislabeled sex, and individuals with high discordance rate to previously genotyped SNPs. In Inter99, a total of 6,161 individuals passed all quality control criteria and the corresponding number was 2,914 for Health2006. The average call rate for all SNPs on the Human Exome BeadChip was 99.0%.

In SHIP-0, samples were genotyped using the Affymetrix Genome-Wide Human SNP Array 6.0. Hybridisation of genomic DNA was done in accordance with the manufacturer’s standard recommendations. The genetic data analysis workflow was created using the Software InforSense. Genetic data were stored using the database Caché (InterSystems). Genotypes were determined using the Birdseed2 clustering algorithm. For quality control purposes, several control samples where added. On the chip level, only subjects with a genotyping rate on QC probesets (QC callrate) of at least 86% were included. Finally, all arrays had a sample callrate > 92%. The overall genotyping efficiency was 98.55.

The genotyped subset of SHIP-TREND was typed using the Illumina Human Omni 2.5 array. Hybridisation of genomic DNA was done in accordance with the manufacturer’s standard recommendations at the Helmholtz Zentrum München. The genetic data analysis workflow and database for storage were similar to that of SHIP-0. Genotypes were determined using the GenomeStudio Genotyping Module v1.0 (GenCall algorithm). All 986 arrays included had a genotyping rate of at least 94%. The overall genotyping efficiency was 99.67 %.

**Supplemental Table 1. Phenotypic characteristics of individuals from Inter99 and Health2006**

|  | **Inter99** | **Health2006** |
| --- | --- | --- |
| n | 5,972 | 3,339 |
| Women, n (%) | 3,050 (51) | 1,842 (55) |
| Age, years | 45 (40-50) | 50 (40-60) |
| Serum vitamin B12, pg/ml  Women, pg/ml  Men, pg/ml | 280 (216-379)  275 (210-378)  286 (221-379) | 379 (307-462)  379 (302-470)  379 (311-455) |
| BMI, kg/m^2^ | 25.6 (23.1-28.6) | 25.2 (22.6-28.2) |
| Smoking, n (%)  Never  Former  Current smoker | 2,080 (35)  1,524 (26)  2,330 (39) | 1,384 (42)  1,079 (33)  843 (26) |
| Alcohol consumption, units per week^a^ | 6.5 (2-14) | 6.0 (2-13) |
| Physical activity, n (%)  0–2 h/week  2–4 h/week  4–7 h/week  7–12 h/week | 677 (12)  1,249 (22)  2,946 (53)  718 (13) | 955 (29)  552 (17)  1,013 (31)  768 (23) |
| Dietary score, n (%)  Unhealthy diet  Moderate healthy diet  Healthy diet | 924 (16)  4,045 (70)  812 (14) | 223 (7)  2,281 (69)  797 (24) |

Only individuals who had serum vitamin B12 levels measured were included. Values are median (IQR) unless otherwise specified.

^a^ 1 unit = 12 g of alcohol

**Supplemental Table 2. Observational association between serum vitamin B12 and BMI**

|  | n | Change in BMI (kg/m^2^) associated with a 20% decrease in serum vitamin B12 (95% CI) | P-value |
| --- | --- | --- | --- |
| Full cohort |  |  |  |
| Adjusted for age and sex |  |  |  |
| Inter99 | 5,969 | 0.10 (0.05;0.14) | 1×10^-4^ |
| Health2006 | 3,336 | 0.19 (0.10;0.27) | 3×10^-5^ |
| Adjusted for age, sex, diet, alcohol consumption, physical activity, and smoking |  |  |  |
| Inter99 | 5,306 | 0.10 (0.05;0.15) | 9×10^-5^ |
| Health2006 | 3,162 | 0.19 (0.10;0.28) | 4×10^-5^ |
| Exclusion of type 2 diabetes patients receiving anti-diabetic treatment |  |  |  |
| Adjusted for age and sex |  |  |  |
| Inter99 | 5,898 | 0.09 (0.05;0.14) | 2×10^-4^ |
| Health2006 | 3,270 | 0.19 (0.10;0.28) | 2×10^-5^ |
| Adjusted for age, sex, diet, alcohol consumption, physical activity, and smoking |  |  |  |
| Inter99 | 5,251 | 0.10 (0.05;0.15) | 9×10^-5^ |
| Health2006 | 3,099 | 0.20 (0.11;0.29) | 2×10^-5^ |

Estimates and P-values are from linear regression.

**Supplemental Table 3. Association between SNPs included in the B12 GRS and serum vitamin B12 levels**

|  |  | Median, pg/ml  (IQR) | | |  | Per allele effect, % (95% CI) | P-value | F-statistic |
| --- | --- | --- | --- | --- | --- | --- | --- | --- |
|  |  | Homozygous for the other allele | Heterozygous | Homozygous for the effect allele | Effect allele frequency, % |  |  |  |
| *MMAA* rs2270655 | n | GG (91%) | GC (9%) | CC (0.2%) | 4.7 |  |  |  |
| Inter99 | 5,747 | 284  (217-383) | 256  (201-338) | 209  (173-266) |  | -11  (-15;-7) | 7×10^-9^ | 34 |
| Health2006 | 2,828 | 384  (310-469) | 342  (282-424) | 323  (277-413) |  | -9  (-13;-5) | 2×10^-6^ | 23 |
| *MUT* rs1141321 |  | CC (38%) | CT (47%) | TT (14%) | 38.1 |  |  |  |
| Inter99 | 5,745 | 291  (224-393) | 277  (212-374) | 266  (208-356) |  | -5  (-6;-3) | 1×10^-8^ | 32 |
| Health2006 | 2,828 | 395  (316-475) | 376  (305-458) | 357  (297-449) |  | -3  (-5;-2) | 2×10^-4^ | 15 |
| *CUBN* rs1801222 |  | GG (40%) | GA (46%) | AA (14%) | 37.2 |  |  |  |
| Inter99 | 5,675 | 296  (226-395) | 277  (214-377) | 248  (193-336) |  | -7  (-9;-5) | 3×10^-17^ | 73 |
| Health2006 | 2,775 | 395  (323-483) | 375  (303-458) | 352  (281-437) |  | -6  (-7;-4) | 2×10^-11^ | 44 |
| *TCN1* rs34324219 |  | CC (79%) | CA (20%) | AA (1%) | 11.2 |  |  |  |
| Inter99 | 5,747 | 288  (221-390) | 254  (198-337) | 218  (172-304) |  | -12  (-15;-10) | 1×10^-23^ | 100 |
| Health2006 | 2,828 | 391  (318-478) | 339  (272-421) | 296  (240-345) |  | -14  (-17;-11) | 6×10^-25^ | 108 |
| *CLYBL* rs41281112 |  | CC (94%) | CT (6%) | TT (0.1%) | 3.0 |  |  |  |
| Inter99 | 5,747 | 282  (217-381) | 258  (195-339) | 148  (138-162) |  | -12  (-16:- 7) | 5×10^-7^ | 25 |
| Health2006 | 2,826 | 382  (310-466) | 350  (273-435) | 261  (245-316) |  | -9  (-14;-5) | 1×10^-4^ | 14 |
| *ABCD4* rs3742801 |  | TT (12%) | TC (45%) | CC (42%) | 64.9 |  |  |  |
| Inter99 | 5,747 | 283  (224-394) | 287  (219-384) | 273  (209-370) |  | -4  (-5;-2) | 2×10^-5^ | 19 |
| Health2006 | 2,828 | 397  (325-490) | 384  (309-461) | 372  (300-461) |  | -3  (-5;-1) | 8×10^-4^ | 12 |
| *CD320* rs2336573 |  | TT (0.1%) | TC (6%) | CC (94%) | 96.9 |  |  |  |
| Inter99 | 5,747 | 670  (508-1,253) | 315  (243-433) | 278  (214-376) |  | -15  (-19;-10) | 8×10^-11^ | 41 |
| Health2006 | 2,828 | 565  (441-689) | 403  (334-472) | 379  (306-464) |  | -7  (-12;2) | 0.007 | 6 |
| *TCN2* rs1131603 |  | CC (0.3%) | CT (11%) | TT (89%) | 94.1 |  |  |  |
| Inter99 | 5,747 | 418  (290-543) | 324  (242-434) | 276  (213-372) |  | -15  (-18;-11) | 3×10^-18^ | 77 |
| Health2006 | 2,828 | 478  (396-539) | 414  (337-501) | 375  (303-459) |  | -10  (-14;-7) | 7×10^-9^ | 33 |
| *FUT6* rs778805 |  | GG (10%) | GA (42%) | AA (48%) | 68.7 |  |  |  |
| Inter99 | 5,745 | 304  (227-397) | 280  (214-379) | 278  (214-373) |  | -3  (-5;-1) | 5×10^-4^ | 12 |
| Health2006 | 2,828 | 387  (330-467) | 381  (308-466) | 379  (303-462) |  | -2  (-4;-0.4) | 0.02 | 5 |
| *FUT2* rs602662 |  | AA (23%) | AG (49%) | GG (28%) | 52.3 |  |  |  |
| Inter99 | 5,747 | 331  (250-438) | 268  (207-359) | 269  (206-353) |  | Add: -10  (-11;-8)  Dom: -20  (-22;-17) | Add: 3×10^-37^  Dom: 1×10^-51^ | Add: 163  Dom: 229 |
| Health2006 | 2,828 | 421  (342-510) | 366  (299-452) | 369  (303-445) |  | Add: -6  (-8;-4)  Dom: -13  (-15;-10) | Add: 1×10^-12^  Dom: 7×10^-19^ | Add:  52  Dom: 82 |

Per allele effects and P-values are from linear regression analyses of the SNPs on log(serum vitamin B12) adjusted for age and sex. F-statistics are calculated using unadjusted linear models. Genotype frequencies and effect allele frequencies (the allele decreasing serum vitamin B12 levels) are stated for Inter99. Add: Additive model; Dom: Dominant model.

**Supplemental Table 4. Association between the B12 GRS and serum vitamin B12 levels**

|  | n | Per allele effect, % (95% CI) | P-value | F-statistic | R^2^, % |
| --- | --- | --- | --- | --- | --- |
| B12 GRS (full-B12 GRS) |  |  |  |  |  |
| Inter99 | 5,671 | -7 (-8;-7) | 5×10^-104^ | 485 | 7.9 |
| Health2006 | 2,773 | -5 (-6;-5) | 3×10^-52^ | 239 | 7.9 |
| B12 GRS excluding *FUT2* rs602662 and *FUT6* rs778805 (B12 GRS) |  |  |  |  |  |
| Inter99 | 5,673 | -7 (-8;-7) | 2×10^-72^ | 331 | 5.5 |
| Health2006 | 2,773 | -6 (-7;-5) | 2×10^-43^ | 197 | 6.7 |

Per allele effects and P-values are from linear regression analyses of the GRS on log(serum vitamin B12) adjusted for age and sex. F-statistics and R^2^ (explained phenotypic variance) are from unadjusted linear modeling.

**Supplemental Table** 5**. Phenotypic characteristics of individuals from SHIP-0 and SHIP-TREND**

|  | **SHIP-0** | **SHIP-TREND** |
| --- | --- | --- |
| n | 2,747 | 4,397 |
| Women, n (%) | 1,414 (51) | 2,267 (52) |
| Age, years | 49 (35-63) | 53 (40-64) |
| Serum vitamin B12, pg/ml  Women, pg/ml  Men, pg/ml | 336 (270;419)  327 (264;409)  345 (276;429) | 453 (352;569)  459 (357; 582)  449 (348;556) |
| BMI, kg/m^2^ | 26.8 (23.6;30.1) | 27.5 (24.5;31.0) |

Values are median (IQR) unless otherwise specified.

**Supplementary Table 6. Association between a genetically induced 20% decrease in vitamin B12 and BMI, examined by two-stage least-squares regression and MR-Egger regression**

|  | Weight, % | Per allele effect, kg/m^2^ (95% CI) | P-value |
| --- | --- | --- | --- |
| **Instrument: Eight vitamin B12 SNPs (excluding *FUT2* and *FUT6*)** | | | |
| Two-stage least-squares regression |  |  |  |
| Inter99 | 59.6 | -0.10 (-0.30;0.09) |  |
| Health2006 | 40.4 | 0.20 (-0.12;0.53) |  |
| Fixed-effect model |  | -0.02 (-0.19;0.14) | 0.78 |
| Heterogeneity: I^2^=60.1%, P =0.11 |  |  |  |
| MR-Egger |  |  |  |
| Inter99 | 61.2 | -0.20 (-0.64;0.24) |  |
| Health2006 | 38.8 | 0.37 (-0.34;1.04) |  |
| Fixed-effect model |  | -0.03 (-0.40;0.39) | 0.87 |
| Heterogeneity: I^2^=46.8%, P =0.17 |  |  |  |
| **Instrument: Ten vitamin B12 SNPs (including *FUT2* and *FUT6*)** | | | |
| Two-stage least-squares regression |  |  |  |
| Inter99 | 65.1 | 0.003 (-0.16;0.16) |  |
| Health2006 | 32.9 | 0.22 (-0.08;0.52) |  |
| Fixed-effect model |  | 0.05 (-0.09;0.19) | 0.47 |
| Heterogeneity: I^2^=37.9%, P =0.20 |  |  |  |
| MR-Egger |  |  |  |
| Inter99 | 60.7 | -0.05 (-0.40;0.31) |  |
| Health2006 | 39.3 | 0.30 (-0.32;0.88) |  |
| Fixed-effect model |  | 0.08 (-0.12;0.36) | 0.58 |
| Heterogeneity: I^2^=25.0%, P =0.25 |  |  |  |

In Inter99, the intercept from the MR-Egger regression was not statistically different from 0, neither when excluding *FUT2* and *FUT6* (P=0.73), nor when including *FUT2* and *FUT6* (P=0.86). Corresponding P-values were 0.63 and 0.75 in Health2006.

*FUT2*: *FUT2* rs602662*. FUT6*: *FUT6* rs778805. 95% CIs were estimated by boot strapping (n=10,000).

**Supplemental Table 7. Test for interaction between the B12 GRS and BMI associated SNPs**

|  | n | P-value |
| --- | --- | --- |
| B12 GRS × BMI GRS |  |  |
| Inter99 | 5,500 | 0.82 |
| Health2006 | 2,633 | 0.43 |
| B12 GRS × *FTO* |  |  |
| Inter99 | 5,505 | 0.86 |
| Health2006 | 2,649 | 0.13 |

B12 GRS: B12 genetic risk score excluding *FUT2* rs602662 and *FUT6* rs778805.

BMI GRS: Genetic risk score based on *FTO* rs9939609, *MC4R* rs17782313, and *TMEM18* rs6548238. *FTO*: *FTO* rs9939609. *FTO* rs9939609, *MC4R* rs17782313, and *TMEM18* rs6548238 were genotyped using the Metabochip as previously described in [1].

***Supplemental Figure 1. Funnel plot.*** Black dots represent causal estimates for each individual SNP based on two-stage least-squares regression (TSLS). Correction for minor allele frequency (MAF) was proportional to the SNP-vitamin B12 standard error. Causal estimates are given for a genetically induced 20% (log_1.2_) decrease in serum vitamin B12.

**References**

1. Sandholt CH, Allin KH, Toft U, Borglykke A, Ribel-Madsen R, Sparso T, et al. The effect of GWAS identified BMI loci on changes in body weight among middle-aged Danes during a five-year period. Obesity (Silver Spring). [Internet]. 2014 [cited 2016 Aug 25];22:901–8. Available from: http://www.ncbi.nlm.nih.gov/pubmed/23804573
